# Integration of Real-Time Image Fusion in the Robotic-Assisted Treatment of Hepatocellular Carcinoma

**DOI:** 10.3390/biology9110397

**Published:** 2020-11-12

**Authors:** Corina Radu, Petra Fisher, Delia Mitrea, Iosif Birlescu, Tiberiu Marita, Flaviu Vancea, Vlad Florian, Cristian Tefas, Radu Badea, Horia Ștefănescu, Sergiu Nedevschi, Doina Pisla, Nadim Al Hajjar

**Affiliations:** 1 Regional Institute of Gastroenterology and Hepatology Prof. Dr. O.Fodor, 400162 Cluj-Napoca, Romania; corina.radu@umfcluj.ro (C.R.); petra.fischer@irgh.ro (P.F.); tefas.cristian@umfcluj.ro (C.T.); horia.stefanescu@irgh.ro (H.Ș.); nadim.alhajjar@umfcluj.ro (N.A.H.); 2 Iuliu Hatieganu University of Medicine and Pharmacy, 400000 Cluj-Napoca, Romania; rbadea@umfcluj.ro; 3 Technical University of Cluj-Napoca, 400114 Cluj-Napoca, Romania; delia.mitrea@cs.utcluj.ro (D.M.); tiberiu.marita@cs.utcluj.ro (T.M.); flaviu.vancea@cs.utcluj.ro (F.V.); vlad.florian@muri.utcluj.ro (V.F.); sergiu.nedevschi@cs.utcluj.ro (S.N.)

**Keywords:** hepatocellular carcinoma, image fusion, ultrasound, targeted treatment, robotic-assisted treatment

## Abstract

**Simple Summary:**

Hepatocellular carcinoma is one of the leading causes of cancer-related deaths worldwide. An image fusion system is developed for the robotic-assisted treatment of hepatocellular carcinoma, which is not only capable of imaging data interpretation and reconstruction, but also automatic tumor detection. The optimization and integration of the image fusion system within a novel robotic system has the potential to demonstrate the feasibility of the robotic-assisted targeted treatment of hepatocellular carcinoma by showing benefits such as precision, patients safety and procedure ergonomics.

**Abstract:**

Hepatocellular carcinoma (HCC) is one of the leading causes of cancer-related deaths worldwide, with its mortality rate correlated with the tumor staging; i.e., early detection and treatment are important factors for the survival rate of patients. This paper presents the development of a novel visualization and detection system for HCC, which is a composing module of a robotic system for the targeted treatment of HCC. The system has two modules, one for the tumor visualization that uses image fusion (IF) between computerized tomography (CT) obtained preoperatively and real-time ultrasound (US), and the second module for HCC automatic detection from CT images. Convolutional neural networks (CNN) are used for the tumor segmentation which were trained using 152 contrast-enhanced CT images. Probabilistic maps are shown as well as 3D representation of HCC within the liver tissue. The development of the visualization and detection system represents a milestone in testing the feasibility of a novel robotic system in the targeted treatment of HCC. Further optimizations are planned for the tumor visualization and detection system with the aim of introducing more relevant functions and increase its accuracy.

## 1. Introduction

Hepatocellular carcinoma (HCC) is the third global leading cause of cancer-related deaths [1]. Ultrasound (US) imaging plays an important role in both the diagnosis and the therapy of HCC; despite the major limitations of US imaging (e.g., diagnostic efficacy is reduced in the case of small, isoechoic lesions or those located subphrenically [2]), it show some important advantages, such as reproducibility, repeatability, low cost and availability. Furthermore, US imaging is used in the treatment of HCC, especially with thermal-based ablative therapies. However, inconspicuous HCC lesions are hard to target on gray scale US, and could therefore negatively affect the outcome of these procedures. In these cases, further imaging using computed tomography (CT) or magnetic resonance imaging (MRI) is required, which leads to increased costs.

Recently, image fusion (IF) systems, which combine real time, gray scale US/CEUS (Contrast-enhanced ultrasound) with multiplanar CT/MRI/PET-CT (Positron emission tomography–computed tomography), were evaluated for the diagnosis and management of HCC. These image guiding systems combine the advantages of multiple medical imaging technologies such as the real-time visualization of planar sections of 3D (tissue) shapes from CT/MRI/PET-CT using US probes by merging the US imaging plane with planes of volumetric information obtained previously (preoperatively). However, IF systems also have limitation, e.g., the volumetric information is static, and dynamic modeling for tissue elasticity, deformation etc., is challenging or slow. IF and the use of US were previously investigated in the scientific literature for various robotic-assisted medical procedures. In [3], the authors present a method of integrating endomicroscopy and US imaging into a single two DoF robotic instrument, providing the surgeon with the possibility to assess superficial tumor margins in high resolution and determining the underlying structure of the tumor with US. Research conducted at the University of British Columbia [4] has proposed a partial augmented reality system with live ultrasound and registered preoperative MRI for guiding robot-assisted radical prostatectomy, while researchers at The Johns Hopkins School of Medicine in Baltimore have researched methods to overcome the limitations of standard TRUS biopsy by implementing a steady and reproducible motion device (i.e., robot) and a tridimensional reconstruction software [5]. Furthermore, the possibility of robotic transrectal ultrasound-guided prostate biopsy has been investigated in [6], using a hands-free probe manipulator, mimicking the same four degrees of freedom that are used manually. Robotic systems (such as [7,8]) may also offer alternative solutions for the image guiding systems using US imaging since the position of the US probe (which is required for an adequate fusion and may be achieved e.g., with electromagnetic tracking [9]) is known via the kinematic models of the robots. In [8], the authors discuss the viability of robotic-assisted brachytherapy and intratumoral-targeted chemotherapy with respect to the technical challenges that the robotic system must overcome in order to ensure patient safety and procedure ergonomics. Although brachytherapy was used alone [10] or in combination with intratumoral chemotherapy [11] for liver cancers treatment, these therapeutic methods are not yet explored (to the authors best knowledge) for the robotic-assisted treatment of HCC.

The aim of this paper is to present the development of a novel detection and visualization system for HCC which is designed for the robotic-assisted targeted treatment of HCC, with a previously introduced robotic system (Pro-Hep-LCT) [7]. The detection and visualization system consists of two main functional modules: one IF module which combines intraoperatory US (due to availability) with preoperative CT imaging, and one automatic HCC detection module able to pinpoint the position and the anatomic details of HCC. The development of the IF system is a milestone for the experimental model of the Pro-Hep-LCT robotic system, which in turn will allow the testing of the feasibility of robotic-assisted treatment of HCC using brachytherapy or intratumoral-targeted chemotherapy.

The paper is structured as follows: Section 2 presents a background of image fusion technologies with respect to the HCC management. Furthermore, Section 2 presents novel robotic systems proposed for the targeted treatment of HCC. Section 3 presents the methods use in the development of the IF system and the system integration into the Pro-Hep-LCT robotic system. Section 4 presents results regarding the IF system, and, finally, the conclusion is presented in Section 5.

## 2. Background

### 2.1. Image Fusiuon Systems—General Considerations

Medical IF is defined as overlaying or spatially matching images from one or more imaging modalities [12]. In order to fuse medical images, a rigid or elastic spatial co-registration of pixels is required. This implies a two-step procedure: image registration and data re-slicing. With rigid co-registration, only translation (panning) and rotation are possible. In the case of elastic co-registration rotation, translation and localized stretching are possible. This improves the matching of anatomical structures [13].

Most systems use rigid transformation matrices (for the dataset correlation between the imaging techniques), since fewer co-registration points are required (when compared to nonrigid transformation matrices). The main limitations of these systems are the absence of compensation for respiration and patient movement, which in most cases cause misalignment. To optimize the alignment of the images, the co-registration should be made in the same respiratory phase as the previously acquired dataset, especially when examining organs that move a lot according to the respiration, for instance, lungs, liver, and spleen. Co-registration can be automatic, using fiducial, external markers, or manual, using anatomical points (landmark) or plane matching (e.g., umbilicus, nipples). The umbilicus or nipple level provide the rough alignment while the matched anatomical points provide the fine-tuning. Specific point matching in the liver often involves the falciform ligament, portal vein, vessel branching points, calcifications, cysts or previously treated lesions [14].

However, fiducial markers are frequently used along with anatomical landmarks to increase the precision of the method. Fiducial markers attached to the body surface around a target organ contain position sensor coils that increase the accuracy of image fusion.

Image-guidance systems based on fiducial registration typically display some measure of registration accuracy. There are three types of errors that are usually used in these analyses: fiducial localization error (FLE), fiducial registration error (FRE) and target registration error (TRE). FLE is the error in localizing the fiducial point, and it is the fundamental cause of the inaccuracy in point-based registration. FRE is the distance between fiducial points after registration. The TRE for a target point is the distance between its registered point and its true corresponding point in the other space. TRE is usually the most clinically relevant error. Since FLE is the basic cause of TRE, estimation of FLE in a single instance of registration is very important in the optimization of point-based registration [15,16].

There are several methods to enable navigation during image-guided procedures with co-display of multiple datasets: electromagnetic and optical tracking provide real-time position data for tracked instruments in a virtual space, while cone beam CT-based navigation permits registration of 3D datasets with fluoroscopy for real-time instrument localization. Most of the systems used for liver imaging and image fusion guided procedures are based on an electromagnetic tracking system. A transmitter and a small sensor are mounted on the US probe providing the position and orientation of the transducer in the spatial volume. The previously recorded CT, MRI or PET/CT dataset is transferred to the US system, and a co-registration from external and internal markers is performed. Afterwards, the CT, MRI, or PET/CT dataset is reformatted in a projection to fit the real-time US image [12].

Regarding ablative therapies of liver tumors, the standard clinical technique involves free hand transcutaneous ultrasonography (TCUS) in conjunction with manual positioning of the tissue ablator. TCUS fails to identify nearly half of all treatable liver lesions, whereas intraoperative or laparoscopic US provides excellent tissue differentiation. Furthermore, freehand manipulation of the US probe critically lacks the level of control, accuracy, and stability required for guiding liver ablation. In response to these limitations, the investigators from Perk Lab at Queens University proposed the use of a fully encoded dexterous robotic arm to manipulate the US probe during surgery. Moreover, investigators proposed a solution of tracking both US and radiofrequency ablation probes for better accuracy in targeting the tumor, reducing the time required for the operation, and minimizing the dependency of the surgical experience [17].

### 2.2. Image Fusion and the Locoregional Therapies of HCC

Locoregional ablative therapies for HCC include curative (thermal ablation) and non-curative (transarterial chemoembolization and transarterial radioembolization) procedures, as well as combinations of these [18]. In most liver interventions, intraoperative ultrasound has become the standard for image guidance. However, ultrasound is a low signal-to-noise imaging modality, and it is difficult to localize the center of a three-dimensional target. Computed tomography (CT) scans or magnetic resonance images (MRI) are also used for image guided procedures. Tumor information determined from CT imaging can be overlaid onto laparoscopic video imaging to allow for more precise resection or ablation [19,20]. Evidence found in the literature suggests that IF significantly improves tumor detection (in contrast to US alone) [21,22], which in turn facilitates accurate targeting and achieves adequate ablative margins. However, Pulse Wave (PW) Doppler or Continuous Wave (CW) Doppler shows better lesion visualization when compared to classic US [23].

Moreover, in [10], the authors reported that Iodine-125 brachytherapy may prolong the progression free survival of patients with residual HCC after being previously treated using RFA. This evidence shows the feasibility of brachytherapy for specific cases of HCC. Furthermore, in [11], the effect of chemoembolization combined with brachytherapy was studied, showing positive effects for the progression free survival. It is also pointed out in [11] that adequate image guidance increase the overall procedure accuracy and patient safety.

### 2.3. Limitations of Image Fusion

Most commercially available IF systems are based on rigid registration, which lacks compensating patient respiration and movement [12]. Since the usual reference datasets (CT, MRI, or PET/CT) are obtained with the patients in a breath-holding state, they contain static images. In comparison, a working dataset (real-time US) is affected by tissue deformation due to the patient’s breathing and movement. Moreover, the position of patients during a real-time US examination can differ from the one during CT/MRI acquisition [24]. In a previous study, the mean maximum registration error between real-time US and fused CT images was 11.5 mm in patients with hepatic metastasis [25].

### 2.4. Robotic-Assisted Targeted HCC Treatment

**Figure 1:**
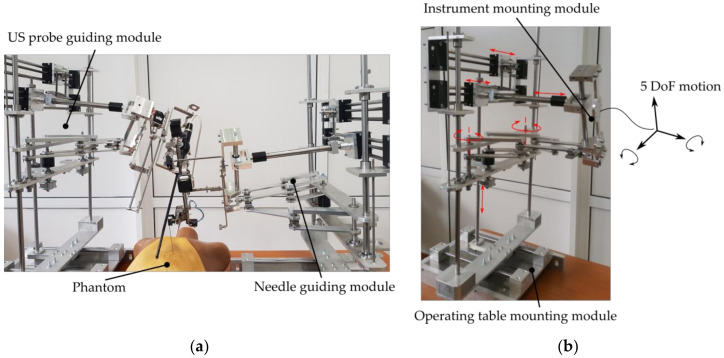
ProHep-LCT robotic system: (**a**) robotic system in “mirrored” configuration; (**b**) one module guiding of the robotic system.

**Figure 2:**
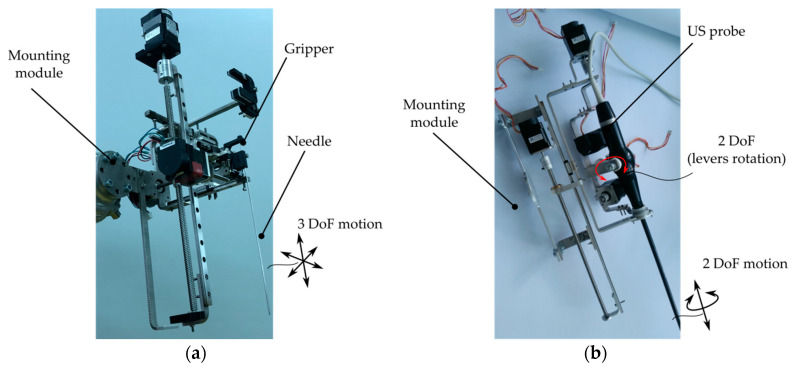

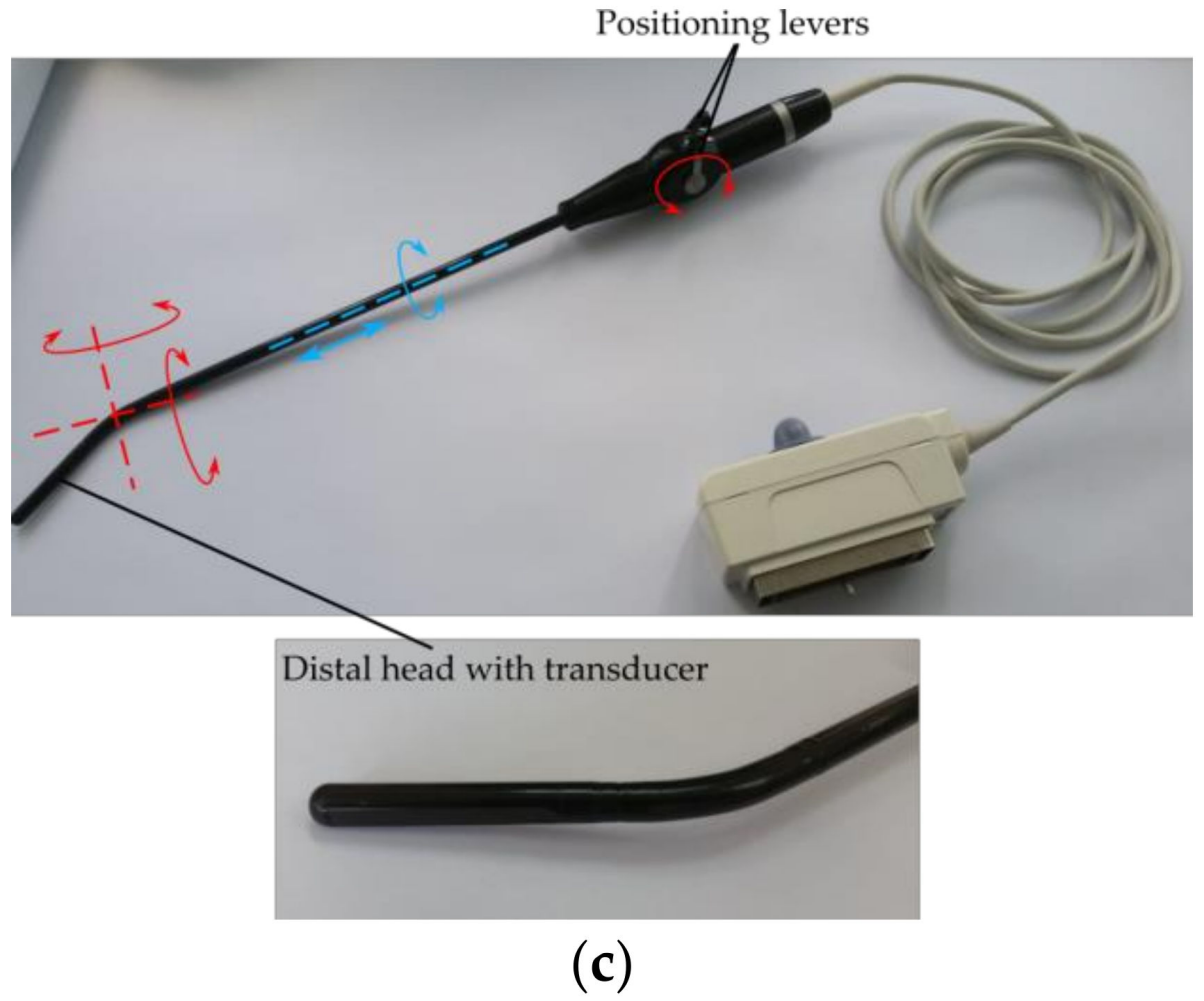
Automated instruments for the ProHep-LCT robotic system: (**a**) needle insertion instrument; (**b**) US probe manipulation instrument; (**c**) Hitachi Arieta intraoperatory US probe.

With respect to the current state of the art regarding the targeted treatment of HCC, in [8], the authors discuss the feasibility of brachytherapy and targeted chemotherapy (i.e., intratumoral delivery of the chemotherapeutic agent such as doxorubicin [26]), proposing technical solutions (robotic systems) that were designed to overcome major technical challenges that these medical procedures impose (accuracy, safety and procedure ergonomics). Moreover, the Pro-Hep-LCT robotic system for the targeted treatment of HCC (using brachytherapy and chemotherapy) was proposed [7], and meticulous studies were performed for its kinematics [7,27,28], workspace optimization (I the medical task), singularities (which have strong implication in the safety operation of robotic systems) [29], and mechanical component optimization (such as mechanical gear optimization [30,31]). ProHep-LCT (patent pending [32]) is an innovative parallel robotic system developed in 2020 by a team of engineers from the Technical University of Cluj-Napoca in collaboration with medical experts from “Iuliu Hatieganu” University of Medicine and Pharmacy from Cluj-Napoca. The robotic system has the following modules (see Figure 1):

- Two identical robotic modules which operate “in mirror” (see Figure 1a where the robotic system is evaluated in laboratory conditions with a phantom). The two modules operate as follows: the first module performs the needle insertion, while the second module guides an intraoperatory US probe that provides visual feedback for tumor location and needle location within the tissue. Each robotic module was designed based on parallel mechanisms to ensure high accuracy (of the treatment delivery), patient safety and procedure ergonomics. Each robotic module has five degrees of freedom (DOF) for guiding the medical instruments (discussed further) in three Cartesian motions and two rotations (Figure 1b).
- Each of the robotic modules are equipped with one of the two novel medical instruments: one multi-needle automated instrument with three DOFs for accurate positioning, insertion, retraction and releasing of specialized needles (for brachytherapy or chemotherapy) (Figure 2a) [33,34], or an automated medical instrument with four DOFs (Figure 2b) which guide a Hitachi Arieta intraoperatory US probe (Figure 2c) for insertion/retraction along the longitudinal axis of the probe, rotation about the longitudinal axis of the probe and two rotations of the distal head about two distinct orthogonal axes [34,35]. Consequently, the needle insertion robotic module has eight DOFs (with three translational redundant ones), whereas the US probe manipulation robotic module has nine DOFs (with two redundant rotations and one redundant translation for fine control of the transducer).
- The Input console: the input console is the master part of the robot control. It is based on a portable computer and was designed to integrate the following components: (1) a graphical user interface with a real-time tumor detection and visualization module with IF (US with CT) received from the 3D reconstruction module, scalable motion for precise medical tool manipulation, and modular control to allow each robotic module to manipulate each automated medical instrument (for needle insertion and for US probe manipulation); (2) a motion input device for real-time continuous control (using a high-precision 3D motion input device) or for setting predefined position (using mouse and keyboard input devices).

## 3. Materials and Methods

### 3.1. Visualization and Detection System Integrated into a Robotic System for the Treatment of HCC

**Figure 3:**
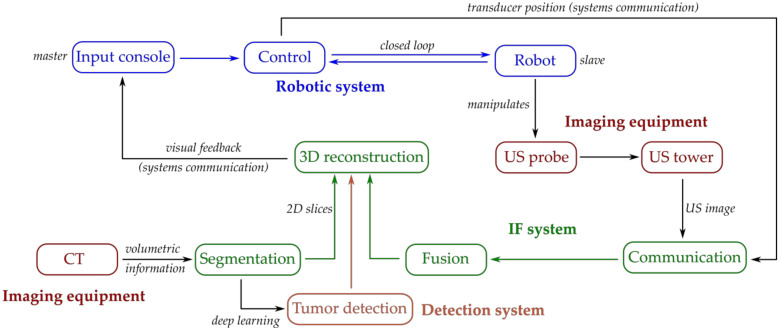
The implementation of the tumor visualization and detection system into the robotic system (general scheme).

Figure 3 presents the general functional scheme which shows the interaction among the robotic system which integrates a novel IF system as well as a tumor detection system. As stated previously the robotic system uses commercially available US imaging equipment as well as specialized needles for the therapeutic agent delivery. The focus hereafter is on the implementation details of the visualization and detection system for HCC.

### 3.2. Computerized System for 3D Reconstruction, Image Fusion and HCC Detection

A computerized IF system was designed at the Technical University of Cluj-Napoca, which aims to perform the automatic detection and 3D reconstruction of HCC and of its anatomical context within the liver. Furthermore, the computerized system is able to highlight the most important blood vessels that possibly intersect a tumor, e.g., the veins from the portal vein and from the lower cavity system, and also some arteries. The IF system is designed for the robotic-assisted targeted treatment of HCC and has the following components [36]:

1. The segmentation module, performing the segmentation (detection and spatial delimitation) of the liver, HCC tumor, and blood vessels using specific methods, such as clustering, region growing, and convolutional neural networks (CNN); this module receives CT images acquired before surgery.

In order to perform HCC fully automated segmentation, both **traditional** and **deep learning** techniques were experimented with, as described below:

a. Traditional methods: In order to achieve HCC segmentation through conventional methods, the fast and robust fuzzy-C-means clustering (FRFCM) technique [36] was adopted. FRFCM assumed a preprocessing phase of morphological reconstruction, and then a fuzzy-C-means clustering algorithm was employed, modified in order to obtain speed improvement. After applying FRFCM, the following were performed during the post-processing phase: image thresholding, the labelling of the resulted objects. The object with the maximum area was selected thereafter, which corresponded to the advanced HCC tumor. The best performance resulted for 10 clusters, employing a squared structural element of size 2 for morphological reconstruction, and a disk structural element of size 3 for the closing operation.
b. Deep learning techniques: Multiple CNN architectures were experimented with, such as ERFNet, EDANet, DeepLabV3 and U-Net [37,38,39]. The ERFNet and EDANet networks were pretrained using traffic data in order to emphasize some basic structures such as edges and curvatures. The DeepLabV3 CNN architecture with a ResNet-101 backbone [38], pre-trained on the Common Objects in Context (COCO) dataset [39], was experimented with in the following situations: (a) on the original images; (b) when the neural network input was composed of three channels, the first receiving the grayscale image as input, the second receiving the morphological reconstruction of the grayscale image and the third, the FRFCM result, after applying the FRFCM technique, with 50 and 100 clusters, respectively. The U-Net CNN architecture, trained from scratch with our CT data, was also employed for HCC segmentation.

2. The tumor detection module: In order to enhance the robotic-assisted treatment modality, an automatic Tumor detection system (using CT images) was also developed, which provides statistical maps (showing the HCC) for visual feedback.
3. The 3D reconstruction module performs 3D reconstruction from the segmented 2D CT images and also generates the 3D anatomic model of HCC within the liver. This module is designed to provide visual feedback to the input console for the medical personnel (e.g., surgeons) operating the robotic system.

The 3D reconstruction module has the following functions: (1) effective 3D reconstruction and volume generation; (2) the application of The Visualization Toolkit (VTK) library filters [40] for highlighting specific regions, such as the white pixels; (3) the integration of the HCC segmentation methods in order to emphasize the tumor within the 3D model of its’ anatomic context; (4) “bounding box” for the 3D volume that allows the user to cut volume sections; (5) re-slice through rotation; and (6) movement of the cross on the screen in order to get variable slices. The last two functionalities are employed in order to generate the 2D slice associated with the current transducer position.

4. The fusion module receives the ultrasound image, as well as the spatial coordinates and orientation corresponding to the current transducer position and emphasizes the corresponding 2D CT slice with the main anatomical elements within the 3D volume.
5. The communication module assures the real-time communication of the computerized system with the robotic system control. The computerized system for image fusion and 3D reconstruction, and the computer application associated with the robot, being situated on different computers, communicate via Ethernet through a socket-based mechanism. Firstly, the image fusion and 3D reconstruction system receives the spatial coordinates and the Euler angles associated with the current ultrasound transducer position from the robot during surgery and, correspondingly, identifies the 2D slice within the 3D anatomic model. Then, the image fusion and 3D reconstruction system provides the robot application with the image of the corresponding 2D section and also the 3D image associated with the 3D anatomic model. Thus, the computerized application for image fusion and 3D reconstruction, as well as the computer application associated with the robot, implement two communication threads, one for the input data and the other for the output data. The computer application associated with the robot finally displays the image corresponding to the 3D anatomic model, as well as that associated with the 2D slice and the ultrasound image that corresponds to the current position of the transducer. The surgeon analyzes these images and, if necessary, applies, with the aid of the robot, the minimum invasive treatment for HCC reduction.

## 4. Results and Discussion

### 4.1. The Dataset

The experimental dataset consisted of 152 contrast enhanced CT images acquired during the arterial phase, belonging to 24 patients affected by HCC. These images were acquired using a Siemens Somatom Perspective machine (2015) with 64 detectors after the injection of the contrast agent. Initially, within these images, the HCC tumors were manually annotated by experienced radiologists in order to provide the ground truth for the automatic segmentation process. As for the segmentation procedures, 60% of the images were included into the training set, 20% of them were included in the validation set, while 20% of these images were included in the test set. Regarding the CNN-based techniques, during the training process, the original images were augmented using random rotation with an angle in the range of −5−5, random translation with offsets sampled from the range −26–26, random scaling between 0.95 and 1.05 of the original image size and random horizontal flipping with a probability of 50%.

### 4.2. Segmentation and Fusion Results

**Figure 4:**
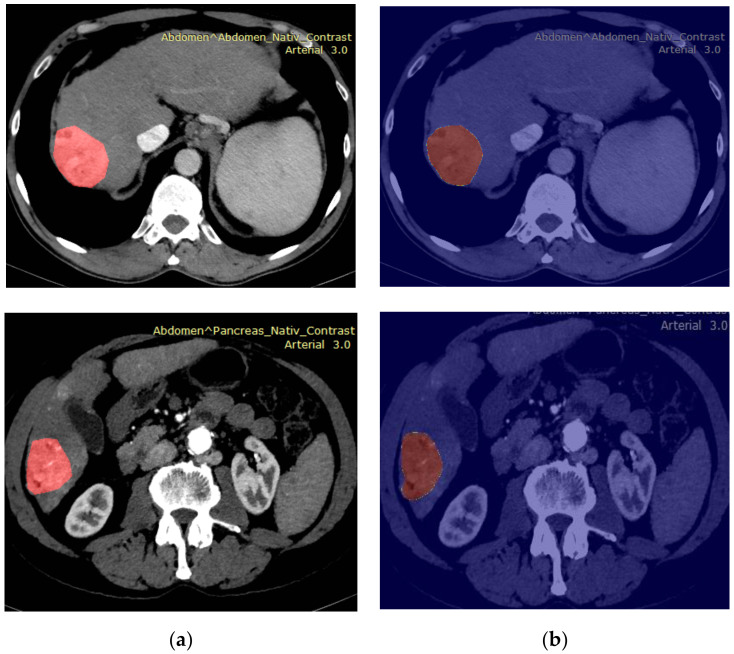
The probabilistic maps and the ground truth for the automatic hepatocellular carcinoma (HCC) segmentation within contrast-enhanced computerized tomography (CT) images: (**a**) the original images with the tumors depicted by the radiologists (the ground truth); (**b**) the automatically generated probabilistic maps (the red pixels denote an increased probability for the pixels that belong to the HCC class, while the blue pixels denote a low probability for the pixels that do not belong to this class).

Concerning the traditional segmentation technique that involves the FRFCM method as described above, the best obtained value for the intersection over union (IoU) metric was 40%. Concerning the deep learning techniques, the CNNs were trained for 150 epochs, the learning rate first being increased from an initial value (2 × 10^−4^) to a maximum value (2 × 10^−3^), then decreasing to 2 × 10^-9^ for each epoch. The ERFNet CNN architecture with an IoU loss function led to an IoU of 70%, which was superior to that provided by the EDANet architecture. The DeepLabV3 CNN architecture with a ResNet-101 backbone [38] provided an IoU of 73.20% in the first case when only the original, unprocessed images were provided at the entrances [36]. The U-Net architecture led to an IoU near 70% as well. Eloquent examples of probabilistic maps, obtained through these methods, are depicted in Figure 4.

**Figure 5:**
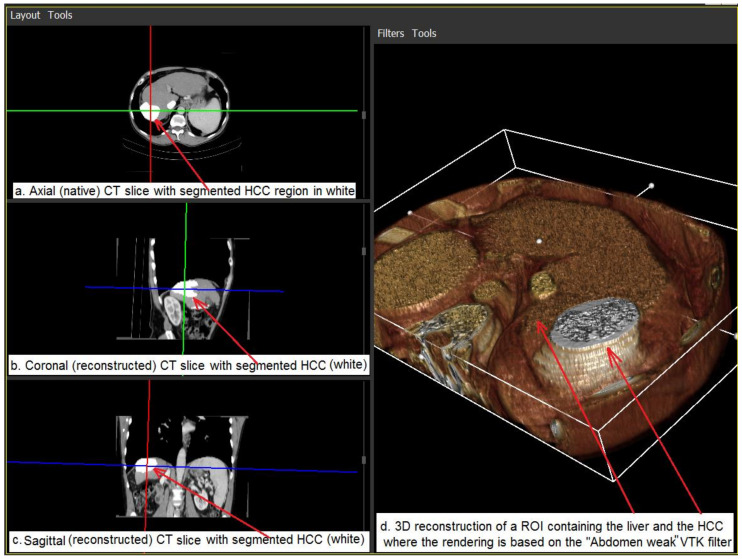
The 3D model of the HCC tumor within the liver (right); the corresponding axial, coronal and sagittal slices with the depicted HCC tumor emphasized (left).

**Figure 6:**
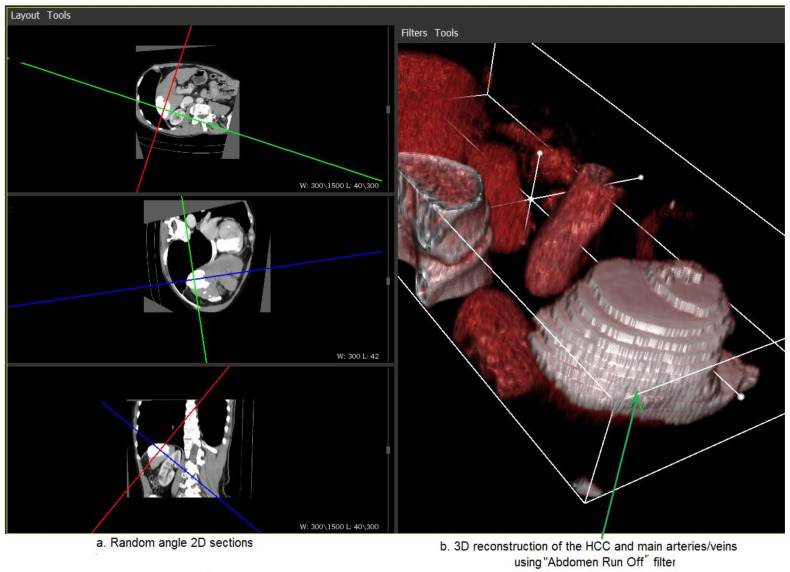
3D representation of the HCC tumor and of the main blood vessels (right); the corresponding random angle 2D sections with the HCC tumor emphasized (left).

According to the previously mentioned results, the best performing deep learning, CNN-based techniques were adopted for HCC segmentation and were integrated within the 3D reconstruction module. Relevant examples are provided in Figure 5 and Figure 6.

Figure 5 illustrates the results of the 3D reconstruction of the regions of interest (ROIs) containing the liver, and the HCCs are shown. The HCC pixels were segmented on the native axial CT scans using a supervised learning approach based on CNN. For visualization purposes, the obtained segmentation masks are overlaid on the native axial scan images in bright (white) intensities. The corresponding coronal and sagittal 2D views and the 3D model of the HCC are reconstructed and rendered using an original VTK [40]-based visualization tool.

Figure 6 illustrates a similar result, but the main blood vessels (arteries and veins) are also highlighted, using a specific VTK filter (the “Abdomen run off” filter) [40]. Moreover, 2D slices corresponding to arbitrary orientations are provided in the left hand side.

### 4.3. Discussion

The targeted treatment of HCC (by means of brachytherapy or chemotherapy) may become a valid therapeutic method if the main challenges that the procedure imposes are addressed, namely, accuracy, safety and ergonomics. On the one hand, accuracy is arguably the most important factor in order to ensure patients’ safety; accurate delivery of the therapeutic agent depends both on the precision of the needle insertion and on the real-time information during the medical procedure [8], a fact that was also pointed out in [11]. On the other hand, the procedure ergonomics is important and has positive effects in diminishing the procedure time and decreasing personnel fatigue. While the robotic systems offer technical solutions for some of the procedure challenges (e.g., increasing accuracy by scalable motion and tremor motion reduction), adequate image guiding may enhance the master–slave control of the robot with real-time visual feedback.

A novel computerized system for 3D visualization and detection of the advanced HCC tumors and of their anatomical context was designed, and it functions in real time. Some similar approaches still exist (see, for example, [41,42]) that either generate the 3D model of the liver, tumor and blood vessels before surgery, using the Myrian-XP-Live application, or perform during surgery the fusion between CEUS and CT images and between CEUS and MRI images, without employing computerized image analysis techniques. However, none of these systems perform both the generation of a preoperative 3D model of the HCC tumor within its’ anatomical context involving advanced segmentation techniques and the application of computerized procedures during surgery in order to emphasize the HCC tumor at a certain moment in two correspondent images of different types (i.e., the 2D CT slice and the ultrasound image, which correspond to the current transducer position).

Future research aims to further improve the segmentation accuracy by gathering more HCC images and using them for the training of CNN structures. Moreover, the optimization of the segmentation process is intended by performing liver detection and by searching the tumor only in the liver region. The CNN-based techniques will be optimized by integrating attention mechanisms, which will enable the detection process to focus on the tumor. The automatic detection and localization of the HCC tumor and of the blood vessels within the ultrasound image can also be performed in the near future, as some preliminary studies and experiments have already been conducted in this direction. The blood vessel segmentation methods are also subjects for future work. Improvement in segmentation accuracy will lead to improvement in the results of the global system. Another future research objective is to effectively combine, through specific computerized procedures, the information from the 2D CT slice, which corresponds to the current position of the ultrasound transducer, with that from the corresponding ultrasound image, acquired during surgery, in order to better highlight the position and contour of the HCC tumor, as well as the blood vessels.

## 5. Conclusions

Image fusion technologies are an extremely useful tool for the interventional hepatologist and radiologist which circumvent some of the limitation of classical US. Moreover, by implementing IF technology for visual feedback on a robotic system (ProHep-LCT), the technical challenges of accuracy and patient safety may be overcome; i.e., despite the accuracy provided by the robotic system for the needle insertion, corrections during the medical act may be necessary, and US alone is not sufficient for a real-time control of the robotic system. Implementing, testing and validating the ProHep-LCT robotic system may offer a window of opportunity in the targeted treatment of HCC.

Further work, necessary for the development of the ProHep-LCT robotic system (with a target of TRL-6), is represented by the following: (1) Further work is required for control development where the implementation of a hybrid control (logic/differential) is desired to increase the patient safety during the procedure. (2) Further work is intended for the visualization and detection system where the detection methods will further be improved (by implementing them on a larger dataset); the blood vessel segmentation method will be fully implemented; and the optimized segmentation methods will be combined with the 3D reconstruction techniques in order to highlight the liver, the HCC tumors and the most significant blood vessels within the 3D volume with maximum accuracy.

## References

[1] Bertuccio P., Turati F., Carioli G., Rodriguez T., La Vecchia C., Malvezzi M., Negri E. (2017). Global trends and predictions in hepatocellular carcinoma mortality. J. Hepatol..

[2] Lee M.W., Kim Y.J., Park H.S., Yu N.C., Jung S.I., Ko S.Y., Hae J.J. (2010). Targeted sonography for small hepatocellular carcinoma discovered by CT or MRI: Factors affecting sonographic detection. Am. J. Roentgenol..

[3] Dwyer G., Giataganas P., Pratt P., Hughes M., Yang G.Z. Miniaturised Robotic Probe for Real-Time Intraoperative Fusion of Ultrasound and Endomicroscopy. Proceedings of the 2015 IEEE International Conference on Robotics and Automation (ICRA).

[4] Samei G., Tsang K., Kesch C., Lobo J., Hor S., Mohareri O., Chang S., Goldenberg S.L., Black P.C., Salcudean S. (2020). A partial augmented reality system with live ultrasound and registered preoperative MRI for guiding robot-assisted radical prostatectomy. Med. Image Anal..

[5] Kaye D.R., Stoianovici D., Han M. (2014). Robotic Ultrasound and Needle Guidance for Prostate Cancer Management: Review of the Contemporary Literature. Curr. Opin. Urol..

[6] Lim S., Jun C., Chang D., Petrisor D., Han M., Stoianovici D. (2019). Robotic Transrectal Ultrasound Guided Prostate Biopsy. IEEE Trans. Biomed. Eng..

[7] Vaida C., Plitea N., Al Hajjar N., Burz A., Graur F., Gherman B., Pisla D. (2020). A new robotic system for minimally invasive treatment of liver tumours. Proc. Rom. Acad. Ser. A Math. Phys. Tech. Sci. Inf. Sci..

[8] Pisla D., Vaida C., Birlescu I., Gherman B., Plitea N. (2020). Risk management for the reliability of robotic assisted treatment of non-resectable liver tumors. Appl. Sci..

[9] Krücker J., Xu S., Venkatesan A., Locklin J.K., Amalou H., Glossop N., Wood B.J. (2011). Clinical utility of real-time fusion guidance for biopsy and ablation. J. Vasc. Interv. Radiol..

[10] Li C., Xihui Y., Dengke Z., Linqiang L., Fazong W., Jianfei T., Jiansong J. (2020). Iodine-125 Brachytherapy Can Prolong Progression-Free Survival of Patients with Locoregional Recurrence and/or Residual Hepatocellular Carcinoma After Radiofrequency Ablation. Cancer Biother. Radiopharm..

[11] Zhiyuan W., Ju G., Wei H., Qingbing W., Ziyin W., Qin L., Jingjing L., Junwei G., Xiaoyi D., Zhongmin W. (2020). Evaluation of doxorubicin-eluting bead transcatheter arterial chemoembolization combined with endovascular brachytherapy for hepatocellular carcinoma with main portal vein tumor thrombus. BMC Cancer.

[12] Ewertsen C., Saftoiu A., Gruionu L.G., Karstrup S., Nielsen M.B. (2013). Real-time image fusion involving diagnostic ultrasound. Am. J. Roentgenol..

[13] Abi-Jaoudeh N., Kruecker J., Kadoury S., Kobeiter H., Venkatesan A.M., Levy E., Wood B.J. (2012). Multimodality image fusion-guided procedures: Technique, accuracy, and applications. Cardiovasc. Intervent. Radiol..

[14] Wood B.J., Kruecker J., Abi-Jaoudeh N., Locklin J.K., Levy E., Xu S., Solbiati L., Kapoor A., Amalou H., Venkatesan A. (2010). Navigation systems for ablation. J. Vasc. Interv. Radiol..

[15] Zhi D. (2015). Towards estimating fiducial localization error of point-based registration in image-guided neurosurgery. Biomed. Mater. Eng..

[16] Fitzpatrick J.M., West J.B., Maurer C.R. (1998). Predicting error in rigid-body point-based registration. IEEE Trans. Med. Imaging..

[17] Boctor E.M., Taylor R.H., Fichtinger G., Choti M.A. (2003). Robotically assisted intraoperative ultrasound with application to ablative therapy of liver cancer. Medical Imaging 2003: Visualization, Image-Guided Procedures, and Display.

[18] Inchingolo R., Posa A., Mariappan M., Spiliopoulos S. (2019). Locoregional treatments for hepatocellular carcinoma: Current evidence and future directions. World J. Gastroenterol..

[19] Galloway R.L. (2001). The process and development of image-guided procedures. Annu. Rev. Biomed. Eng..

[20] Herline A., Stefansic J.D., Debelak J., Galloway R.L., Chapman W.C. (2000). Technical advances toward interactive image-guided laparoscopic surgery. Surg. Endosc..

[21] Ahn S.J., Lee J.M., Lee D.H., Lee S.M., Yoon J.H., Kim Y.J., Lee J.H., Yu S.U., Han J.K. (2017). Real-time US-CT/MR fusion imaging for percutaneous radiofrequency ablation of hepatocellular carcinoma. J. Hepatol..

[22] Lee J.Y., Choi B.I., Chung Y.E., Kim M.W., Kim S.H., Han J.K. (2012). Clinical value of CT/MR-US fusion imaging for radiofrequency ablation of hepatic nodules. Eur. J. Radiol..

[23] Rafailidis V., Sidhu P.S. (2020). Ultrasound of the Liver. Imaging of the Liver and Intra-Hepatic Biliary Tract. Medical Radiology.

[24] Lee M.W. (2014). Fusion imaging of real-time ultrasonography with CT or MRI for hepatic intervention. Ultrasonography.

[25] Hakime A., Deschamps F., De Carvalho E.G.M., Teriitehau C., Auperin A., De Baere T. (2011). Clinical evaluation of spatial accuracy of a fusion imaging technique combining previously acquired computed tomography and real-time ultrasound for imaging of liver metastases. Cardiovasc. Intervent. Radiol..

[26] Solorio L., Wu H., Hernandez C., Gangolli M., Exner A.A. (2016). Ultrasound-guided intratumoral delivery of doxorubicin from in situ forming implants in a hepatocellular carcinoma model. Ther. Deliv..

[27] Birlescu I., Husty M., Vaida C., Gherman B., Tucan P., Pisla D. (2020). Joint-Space Characterization of a Medical Parallel Robot Based on a Dual Quaternion Representation of SE(3). Mathematics.

[28] Vaida C., Tucan P., Plitea N., Lazar V., Al Hajjar N., Pisla D. (2019). Kinematic analysis of a new parallel robotic system for minimally invasive therapy of non-resecable hepatic tumors. IFToMM World Congress on Mechanism and Machine Science.

[29] Birlescu I., Husty M., Vaida C., Plitea N., Nayak A., Pisla D. (2019). Complete Geometric Analysis Using the Study SE(3) Parameters for a Novel, Minimally Invasive Robot Used in Liver Cancer Treatment. Symmetry.

[30] Antal A.T., Antal A. (2010). Helical gear dimensions in the case of the minimal equalized specific sliding. Proceedings of the SYROM 2009—10th IFToMM International Symposium on Science of Mechanisms and Machines.

[31] Antal A.T. (2015). Addendum modification of spur gears with equalized efficiency at the points where the meshing stars and ends. Mechanika.

[32] Plitea N., Pisla D., Vaida C., Gherman B., Tucan P. (2018). PRoHep-LCT-Parallel robot for the minimally invasive treatment of hepatic carcinoma. Patent Pending A.

[33] Gherman B., Birlescu I., Burz A., Pisla D. (2019). Automated medical instrument for the insertion of brachytherapy needles on parallel trajectories. Patent Pending A.

[34] Gherman B., Birlescu I., Burz A., Pisla D. (2020). Kinematic analysis of two innovative medical instruments for the robotic assisted treatment of non-resectable liver tumors. EuCoMeS 2020: New Trends in Mechanism and Machine Science.

[35] Birlescu I., Vaida C., Gherman B., Burz A., Tucan P., Plitea N., Pisla D. (2019). Automated medical instrument for ultrasound laparoscopic probe guiding. Patent Pending A.

[36] Mitrea D., Marita T., Vancea F., Nedevschi S., Mitrea P., Neamt G.M., Timoftei S., Florian V., Pisla D., Radu C. (2020). Towards building a computerized system for modelling advanced HCC tumors, in order to assist their minimum invasive surgical treatment. New Trends in Mechanisms and Machine Science, the 8th European Conference on Mechanism Science (EuCoMeS).

[37] Chen L., Papandreou G., Schroff F., Adam H. (2018). Rethinking Atrous Convolution for Semantic Image Segmentation. arXiv.

[38] Christ P.F., Ettlinger F., Grün F., Elshaera M.E., Lipkova J., Schlecht S., Ahmaddy F., Tatavarty S., Bickel M., Bilic P. (2017). Automatic liver and tumor segmentation of CT and MRI volumes using cascaded fully convolutional neural networks. arXiv.

[39] Smith L.N., Topin N. (2017). Super-Convergence: Very Fast Training of Residual Networks Using Large Learning Rates. arXiv Prepr..

[40] Schroeder W., Martin K., Lorensen B. The Visualization Toolkit: An Object-Oriented Approach to 3D Graphics, 4th ed. http://www.kitware.com.

[41] Gong Y., Tang Y., Geng Y., Zhou Y., Yu M., Huang B., Sun Z., Tang H., Jian Z., Hou B. (2019). Comparative safety and effectiveness of ultrasound guided radiofrequency ablation combined with preoperative three-dimensional reconstruction versus surgical resection for solitary hepatocellular carcinoma of 3–5 cm. J. Cancer.

[42] Li K., Su Z., Xu E., Huang Q., Zeng Q., Zheng R. (2017). Evaluation of the ablation margin of hepatocellular carcinoma using CEUS-CT/MR image fusion in a phantom model and in patients. BMC Cancer.

